# Vaginal Natural Orifice Transluminal Endoscopic Surgery Hysterectomy Deconstructed: Expanding Minimally Invasive Gynecologic Surgeons' Toolbox 

**DOI:** 10.1089/gyn.2023.0098

**Published:** 2024-04-12

**Authors:** Veronica Lerner, Andrea E Stuart, Jan Baekalandt

**Affiliations:** ^1^ Department of Obstetrics & Gynecology, Lenox Hill Hospital, Zucker School of Medicine at Hofstra/Northwell Health, Hempstead, New York, USA.; ^2^ Department of Obstetrics and Gynaecology, Institution of Clinical Sciences, Lund University, Lund, Sweden.; ^3^ Department of Obstetrics and Gynaecology, Helsingborg Hospital, Sweden.; ^4^ Department of Gynaecologic Oncology, Imelda Hospital, Bonheiden, Belgium.

**Keywords:** vNOTES hysterectomy, vaginal natural orifice transluminal endoscopic surgery, minimally invasive gynecologic surgery, minimally invasive hysterectomy

## Abstract

**Background:**

The introduction of vaginal natural orifice transluminal endoscopic surgery (vNOTES) to the toolbox of gynecologic surgeons has the potential to reverse the trend of vaginal hysterectomy declines.

**Methods:**

This review discusses nuances of the vNOTES technique applied to hysterectomy; describes vNOTES hysterectomy, step-by-step (including tips and tricks for low- and high-complexity cases for surgeons who may want to incorporate vNOTES hysterectomy into their surgical repertoires); and examines evidence and research trends in this field.

**Results:**

The descriptions in the text, figures, tables, and videos all contribute to giving readers a clear understanding of vNOTES, its advantages, limitations, and research potentials.

**Conclusions:**

vNOTES hysterectomy is a unique blend of vaginal, laparoscopic, and laparoendoscopic single-site surgery (LESS) techniques and is not a new procedure, but rather another tool to use in minimally invasive gynecologic surgery. (J GYNECOL SURG 40:78)

## Introduction

Before the emergence of laparoscopy, when open abdominal and vaginal hysterectomies were the only options, a large proportion of hysterectomies had the potential to be done vaginally. However, multiple barriers prevented widespread use of the vaginal route.^1,2^ The history of the vaginal natural orifice transluminal endoscopic surgery (vNOTES) evolution is tied closely to transabdominal laparoendoscopic single-site surgery (LESS),^3^ which emerged as an alternative to popular multiport laparoscopy and robotics. However, the challenges of transabdominal LESS halted its widespread adoption,^4^ and vNOTES emerged in the last decade as a way to revive vaginal surgery, which has been declining at a rapid rate, while laparoscopy and robotics gained acceptance.

This expert review (1) discusses nuances of the vNOTES technique as it applies to hysterectomy; (2) describes vNOTES hysterectomy, step-by-step, including tips and tricks for low- and high-complexity cases for surgeons who may want to incorporate vNOTES hysterectomy into their surgical repertoires; and (3) examines evidence and research trends in this field.

## Patient Selection

Indications and contraindications to vNOTES hysterectomy as well as considerations for preoperative planning are listed in Table 1.^5,6^ While it is beyond the scope of this article to address the use of the vNOTES route for endometriosis, exploration of this route continues; however, the surgical community is not yet ready for wide adoption of this technique.^7^

**Table 1.** Patient Selection for Vaginal Natural Orifice Transluminal Endoscopic Surgery Hysterectomy

Factors	Rationale	Preoperative evaluation	Surgical considerations
**Contraindications**			
Obliterated posterior cul-de-sac	• Inability to enter the peritoneal cavity that prevents inner-ring placement & ability to initiate laparoscopic part of vNOTES • Inability to see if the colon is adherent to the posterior uterus, which puts the patient at risk for bowel injury at the time of entry	• Extensive history taking with a focus on pelvic pain & endometriosis symptoms • Obtaining the most accurate surgical history possible, including operative reports, hospitalization records & treatment history • Preoperative examination & examination under anesthesia at the beginning of the case with suspicion of uterosacral & rectovaginal nodularity, limited mobility & adhesions • Advanced imaging, such as MRI, protocoled for endometriosis or dynamic pelvic US performed by a sonologist	• Conditions that might cause obliteration include endometriosis, severe PID, pelvic radiation & colorectal surgery such as low anterior resection • Best practice to address obliteration is to perform adhesiolysis via a transabdominal laparoscopic approach
Endometriosis	• Standard-of-care is to excise disease via transabdominal laparoscopic or robotic approach	• Extensive history taking with a focus on pelvic pain & endometriosis symptoms • Preoperative examination & examination under anesthesia at the beginning of the case with suspicion of uterosacral & rectovaginal nodularity, limited mobility & adhesions • Advanced imaging, such as MRI, protocoled for endometriosis or dynamic pelvic ultrasound performed by a sonologist	• Robotic platforms & various surgical approaches are being investigated to be able to perform endometriosis excision, including sidewall & rectovaginal lesions; these techniques are not ready for widespread clinical adoption
Severe distortion of pelvic sidewall anatomy due to fibroids	• Cervical, lower uterine segment, broad ligament fibroids & fibroids extending into the sidewall prevent surgeon from visualizing uterine arteries at the site of the transection & limit ability to isolate them from sidewall • In cases of advanced pathology requiring extensive ureterolysis and sidewall dissection, transabdominal laparoscopic & robotic approaches are required	• History suggestive of sidewall compression (asymmetric lymphedema & unilateral hip & pelvic floor pain) • Preoperative examination & examination under anesthesia indicating sidewall involvement with limited mobility & minimal space between fibroids in lower uterine segment & lateral sidewall • Advanced imaging, such as MRI, protocoled or dynamic pelvic US performed by a sonologist, close collaborative relationship with radiologists and sonologists & case-by-case treatment planning in a multidisciplinary setting	• Need to be able to secure uterine blood supply by visualizing & transecting uterine arteries at the beginning of laparoscopic part • If able to secure uterine blood supply on 1 side, surgeon can detach entire side to rotate the uterus internally & obtain adequate exposure to transect uterine arteries on the contralateral more challenging side • Fibroids can be dissected out of the sidewall with adequate visualization of the ureter, vessels & all sidewall structures, but that depends on ability to see those structures, dissect & secure uterine arteries, and on surgeon's skill set

**Indications**			
AUB	• Benefits of vaginal route	• Follow standard evaluation protocols	• Contained tissue extraction is an option in case of enlarged uterus & suspicion for hyperplasia
Fibroids	• Benefits of vaginal route • “Ideal” fibroid distribution would be such that the cervix & lower uterine segment are not involved & the uterus has a shape of a “mushroom”—narrow where uterine blood supply comes in with adequate lateral sidewall access that, in turn, would enable surgeon to secure the uterine blood supply	• History suggestive of sidewall compression (asymmetric lymphedema & unilateral hip & pelvic floor pain) • Preoperative examination & examination under anesthesia indicating sidewall involvement with limited mobility & minimal space between fibroids in the lower uterine segment & lateral sidewall • If sidewall involvement is suspected, advanced imaging, such as MRI, protocoled or dynamic pelvic US performed by a sinologist should be considered; close collaborative relationship with radiologists & sonologists & case-by-case treatment planning in a multidisciplinary setting	• No “size” cutoff exists clinically as long as physical examination & imaging are consistent with the ability to access uterine blood supply • Uterus can be rotated in the pelvis sequentially into a more favorable orientation to improve exposure as hysterectomy proceeds (example: 1 “easier” side of the hysterectomy can be completed first to gain access to the more-difficult side) • All blood supply is secured before specimen extraction begins; this decreases blood loss
Adenomyosis	• Benefits of vaginal route	• Extensive history taking with a focus on pelvic pain & endometriosis symptoms • Preoperative examination & examination under anesthesia at the beginning of the case with suspicion of uterosacral & rectovaginal nodularity, limited mobility & adhesions • Advanced imaging, such as MRI, protocoled for endometriosis or dynamic pelvic US performed by a sonologist	• If clinically suspicious of comorbid endometriosis, transabdominal approach is standard-of-care to perform endometriosis excision at the time of hysterectomy

**Patient factors**			
C-section(s) scar	• Adhesiolysis can be started during vaginal part & completed during laparoscopic part	• Preoperative examination enables surgeon access to extent of adhesions	• Laparoscopic portion of vNOTES adhesiolysis enables surgeon to reach further cephalad & perform more extensive manipulation, compared to conventional vaginal route, enabling surgeon to address more extensive adhesions & convert transabdominal cases to vNOTES route • Inner ring is placed between the colpotomy incision on the vaginal epithelium & C-section scar adhesions and peritoneum • Using “lateral-to-medial” technique by securing uterine blood supply first, then lysing adhesions from lateral–to–medial to reflect ureters, minimize bleeding & decrease bladder injury risk • Anterior peritoneum is blue or gray because it's translucent, unlike the dense white scar; also, peritoneum moves in & out slightly from intermittent insufflation of pneumoperitoneum or patient ventilation, showing “wave” or the “sail” sign • Can retrograde fill the bladder, but that is rarely necessary due to “upside down” view of bladder, which is retracted by the inner ring
Abdominal wall mesh from prior surgery	Mesh avoidance	• Preoperative evaluation, including prior operative reports & imaging	• Vaginal route enables surgeon to avoid having to place ports & navigate around the mesh or having to cut through it
Adhesions in the mid-& upper-abdomen, multiple prior abdominal surgeries	Avoid adhesiolysis	• Preoperative evaluation, including prior operative reports	• Vaginal route enables surgeon avoid having to place ports & navigate around the adhesions &/or having to perform extensive adhesiolysis
Rigid abdomen, resulting in limited insufflation (nulliparity, abdominoplasty)	• Benefits of vaginal route	—	• Low pressures (8–10 mm Hg) are sufficient for visualization, because most of the surgical work occurs in the pelvis, which is a rigid bony structure & does not need to be insufflated to the same pressures as the transabdominal approach to obtain adequate visualization • Laparotomy pad or 4 x 4 sponge inserted into the pelvis at the beginning of the case helps bowel retraction and visualization
Limited vaginal access	Conditions that contribute to narrow introitus & vaginal canal (examples: obesity, nulliparity, postmenopausal status & gender-affirming surgery) limit ability to perform conventional vaginal hysterectomy	—	• Compared to conventional vaginal hysterectomy, in which limited access may preclude ability to complete entire case, with vNOTES, surgeon only needs to be able to perform colpotomies & place vNOTES port, to complete procedure laparoscopically • Improved access enables the surgeon to convert transabdominal cases to the vNOTES route • For obese patients, vNOTES bypasses challenges of addressing abdominal fat, enables shortest distance to target anatomy & requires less Trendelenburg positioning, as laparotomy pads can be used to move intestines out of the surgical field & lower insufflation pressures
Cosmesis	• Avoidance of abdominal incisions is integral part of patient-centered care, wherein patient preference is considered		• Special consideration for gender-affirming hysterectomies wherein abdominal incisions could worsen gender dysphoria

vNOTES, vaginal natural orifice transluminal endoscopic surgery; MRI, magnetic resonance imaging; US, ultrasound; PID, pelvic inflammatory disease; AUB, abnormal uterine bleeding; C-section, cesarean section.

The ideal candidate for low-complexity vNOTES hysterectomy is a patient with abnormal uterine bleeding with or without fibroids. There is no “size” cutoff as long as the physical examination and imaging are consistent with the above description. Uterine weight over 1 kg is commonplace and vNOTES hysterectomy over 3 kg has been documented.^8–10^ Adenomyosis is also often addressed via vNOTES hysterectomy; however, the possibility of coexisting endometriosis needs to be considered due to its high prevalence in this patient population. Surgical teams must have close collaborative relationships with radiologists and sonologists.^11^

The “bottom-up” approach to anatomy and pathology offers several advantages. The surgeon controls the uterine blood supply at the beginning of the case, not at the end of it.^5^ Bladder and ureters are mobilized early in the case, not later. While the incidence of bladder and ureteral injuries varies widely, as it depends on patient selection, practice setting, and surgeon skill set, this could explain why ureteral injuries have been less common in the vaginal compared to the transabdominal minimally invasive gynecologic surgery (MIGS) approach in some studies.^12,13^

In addition, the bottom-up approach is advantageous in cases requiring cesarian section scar adhesiolysis or cases of large fibroid burden (Videos 1 and 2).^14,15^ Larger studies and systematic reviews have shown that the vaginal route is most favorable in terms of reducing complications.^16,17^ Among surgeons with the highest level of skill (fellowship training and high volume) limited to a specific route, the risk of urinary-tract injury during a hysterectomy drops from 1% to 0.04%.^18^ This highlights the urgent need for research on high-quality gynecologic surgical outcomes focused on nuanced patient- and surgeon-specific factors.

**FIG. 1.**
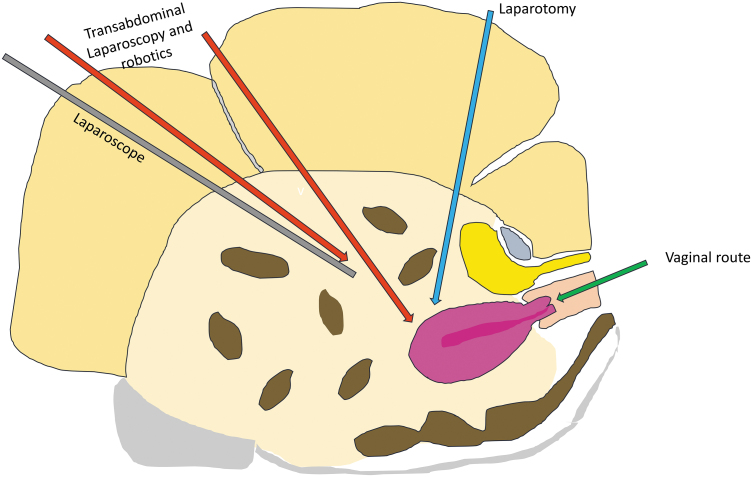
Vaginal natural orifice transluminal endoscopic surgery approach to hysterectomy in comparison to transabdominal laparoscopy and robotics in obese patients. Color images are available online.

For obese patients, vNOTES, just like conventional vaginal hysterectomy, offers several advantages in comparison to the transabdominal route (Fig. 1).

The decision-making process of a surgeon who opts to keep or add a vaginal approach to the surgical repertoire differs from that of a surgeon limited to the transabdominal laparoscopic or robotic route. First, a vNOTES surgeon must go through the preoperative evaluation required during patient selection to triage the patient to the appropriate route. Second, a vNOTES surgeon needs to have a lower threshold for a higher conversion rate to laparoscopy, compared to a surgeon whose only route is a transabdominal route, when conversion means a laparotomy. In general, all surgeons will have a conversion rate that depends on their skill set, pathology, patient factors, and resources in the surgeon's local practice settings. All surgeons strive to minimize that rate, as conversions prolong operating room time and increase complication rates. However, when a vaginal route is converted to a transabdominal laparoscopic or robotic route, this conversion remains within the minimally invasive realm.

When discussing the vaginal versus the transabdominal MIGS approach with patients, it is important to explain the benefits of avoiding the transabdominal route: less pain and no abdominal incisions; avoidance of the risk of injury at the time of laparoscopic entry; avoidance of nerve injury and nerve entrapment; and avoidance of incisional hernias (in case of minilaparotomy when transabdominal tissue extraction is utilized).

Other factors, such as patient and surgeon preferences, surgeon skill, learning curves, teaching trainees, implementation in different practice settings, and adoption rates are beyond the scope of this review.

## vNOTES Hysterectomy Surgical Technique

**FIG. 2.**
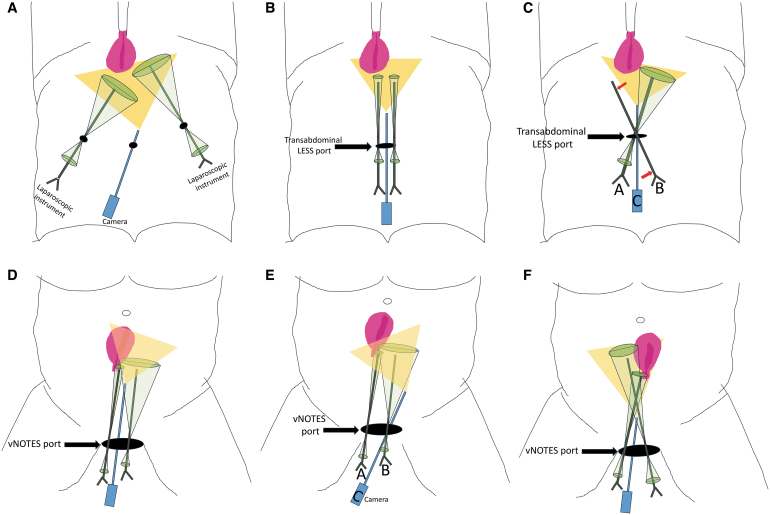
Comparison of multiport, transabdominal laparoendoscopic single-site surgery (LESS) and vaginal natural orifice transluminal endoscopic surgery (vNOTES) techniques. **(A)** Multiport transabdominal laparoscopy. The *green oval shadows* represent movements of the instruments and the *yellow triangle* is the field of view (FOV) from the camera. The instruments and camera each have a wide range of movement (ROM) and the working space is less limited. **(B)** Transabdominal LESS parallel technique. The *green oval shadows* represent movements of the instruments, the *yellow triangle* is the FOV from the camera. The instruments and camera each have a limited ROM and working space is limited. **(C)** Transabdominal LESS cross technique. The *green oval shadow*s represent movements of the instruments, the *yellow triangle* is the FOV from the camera. *Red arrows* show the technique of keeping 1 instrument static *(instrument A)* to retract, which enables a greater ROM for *instrument B* with a *green oval shadow*, compared to the parallel technique. *C* is the camera. **(D)** vNOTES hysterectomy, parallel technique with 0° laparoscope. The *green oval shadows* represent the movements of the instruments, the *yellow triangle* is the FOV from the camera. The instruments and camera each have a wide ROM and working space is less limited in comparison to transabdominal LESS. **(E)** vNOTES hysterectomy, parallel technique with 30° laparoscope. The *green oval shadows* represent movements of the instruments, the *yellow triangle* is the FOV from the camera. The instruments and camera each have a wide ROM in comparison to transabdominal LESS. The use of a 30° laparoscope improves visualization of the target anatomy in comparison to a 0° laparoscope; however, its use of the 30° laparoscope is not essential in lower-complexity cases. **(F)** vNOTES hysterectomy cross technique with 30° laparoscope. The *green oval shadows* represent movement of the instruments, the *yellow triangle* is the FOV from the camera. The instruments and camera each have a wider ROM and working space is less limited. As surgery progresses from caudad to cephalad, movements become more restrictive and working space becomes more limited. Cross-technique and use of a 30° laparoscope improve visualization and increase working space. The primary surgeon orients their hands horizontally in order to minimize external collisions. Color images are available online.

**FIG. 3.**
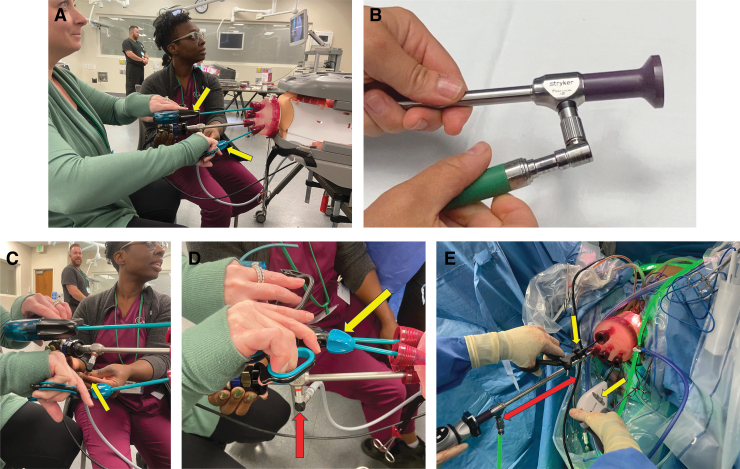
Optimizing working space by minimizing external collisions. **(A)** The primary surgeon orients her hands horizontally in order to minimize external collisions with the instruments and the camera and to maximize external working space while performing a vaginal natural orifice transluminal endoscopic surgery (vNOTES) hysterectomy during a simulation. Both laparoscopic instruments have handles each with a rotation knob that enables 360° rotation of the instrument tips (*yellow arrows*). **(B)** A right-angle light cord adapter is used to keep the light cord out of the working space. **(C)** The primary surgeon orients her hands horizontally and staggers her instruments while performing a vNOTES hysterectomy during a simulation. However, the camera has not been moved out of the way of the laparoscopic instruments and is colliding with the surgeon's right hand (*yellow arrow*), although the right-angle adapter is used to keep the light cord out of the working space. **(D)** The primary surgeon orients her hands horizontally and staggers her instruments (with her right hand slightly ahead of her left hand) to minimize external collisions and maximize the external working space while performing a vNOTES hysterectomy during a simulation. The camera has been moved out of the way of the laparoscopic instruments with the light post placed at the 6 o'clock position. The *yellow arrow* points to the rotation knob that enables a 360° rotation of the instrument tips. The *red arrow* points to the right-angle light adapter. **(E)** Bariatric length scope and instruments. To decrease the likelihood of external collisions further, a bariatric length laparoscope is used, which moves the camera head out of the way for the instrument in the surgeon's left hand (*red arrow*), which is oriented horizontally. Both laparoscopic instruments have rotation knobs that enable 360° rotation of the instrument tips (*yellow arrows*). Color images are available online.

Learning the technique of vNOTES hysterectomy requires adoption of LESS techniques while continuing to utilize the conventional vaginal surgery skill set. Tables 2 and 3, Figures 2 and 3, and Videos 1–8 describe vNOTES hysterectomy steps and techniques in detail. Preparation, setup, and instrumentation were described in a 2022 article.^1^ It is beyond the scope of this review to cover the vaginal portion of this procedure for novice surgeons. Numerous resources (textbooks, articles, surgical videos) are available on this topic if more indepth understanding is desired.^19,20^

**Table 2.** Vaginal Natural Orifice Transluminal Endoscopic Surgery (vNOTES) techniques applied to vNOTES hysterectomy

Surgical technique aspects	Tips & tricks
**LESS port**	
Restricted instrument movement	• In vNOTES, the access point is the colpotomy, which results in a wider, less-rigid aperture & less movement restriction, compared to transabdominal LESS route. • Hysterectomy is less restrictive than surgery via posterior colpotomy (such as adnexal surgery & hysteropexy). When learning vNOTES, hysterectomy is a recommended procedure of choice over adnexectomy. • During a hysterectomy, as the surgeon moves more cephalad along the broad ligament, the inner ring opens more, resulting in less restriction from the inner ring. • Working in the distal pelvis results in less restriction than proximally (for example, the board ligament is easier to triangulate than the infundibulopelvic ligament near the pelvic brim)
Choice of inner-ring size	• The vast majority of hysterectomies are best performed with GelPOINT V-Path (Applied Medical, Rancho Santa Margarita, CA, USA) size 9.5 regardless of parity, anatomy & indication for surgery. • In a rare case of a very narrow vaginal canal & vaginal apex (as in some nulliparous & gender-affirming surgery patients & patients not having vaginal penetrative sex) this movement restriction might be more noticeable due to the more-narrow access point. In addition, consider using a smaller inner ring to facilitate ring placement (V-Path size 7). • In contrast, a wide vaginal canal in some multiparous & prolapse patients might result in expulsion of V-Path 9.5 inner ring. Consider using a larger ring to avoid expulsion (V-Path size 11).
Internal & external instrument collisions (“sword fighting”)	• Stagger instruments & camera by utilizing a bariatric-length scope & a long bariatric-length bipolar device. • Utilize right-angle light-source adaptor, which keeps the light cord out of the working space. • Orient hands horizontally rather than vertically. • Use straight instruments with articulating handles, preferably those that can rotate >360°. • In vNOTES, the access point is the colpotomy, which results in a wider, less-rigid aperture which reduce instrument collisions, compared to a transabdominal LESS route.
Loss of triangulation with straight instruments	• In vNOTES, the access point is the colpotomy, which results in a wider, less-rigid aperture that reduces loss of triangulation, compared to a transabdominal LESS route. • Parallel versus cross technique is utilized depending on circumstances.
Limited FOV	• Use a 30° scope instead of 0° scope. • Surgeon driving the camera follows the instruments rather than directing where instruments need to go. • Start at the “home position” with instruments inserted under direct visualization & positioned slightly ahead of the camera. • Introduce instruments under direct visualization at all times to avoid visceral injuries; this might require person diving the camera to back up to the home position during instrument exchange. • The default position for the camera is “looking up” with the light post at the 6 o'clock position. • As surgery progresses, light-post movement between 4 & 8 o'clock is sufficient to visualize most structures during a hysterectomy. • Be sure to insert a Foley catheter & keep it in for the duration of the case for drainage, as the catheter keeps the bladder out of the FOV.
Smoke evacuation	• In LESS, insufflation port & smoke evacuation ports are located in closer proximity to each other, compared to a transabdominal multiport configuration. As a result, smoke generated in the field tends to “float” & linger near the camera port, obscuring the view. In addition, cold gas from the insufflator could cloud the lens because of condensation. • A vNOTES route results in smoke floating cephalad & dispersing in the abdomen, rather than concentrating near the lens. • Use the AirSeal^®^ insufflation system (ConMed, Largo, FL, USA) by placing the AirSeal^R^ port into the gel (off-label use). • Insert an arterial line catheter connected to the smoke-evacuation tubing, thread the catheter into the vagina external to the rings & place it in the pelvis at the desired site. • Choose a bipolar device with minimal smoke generation.
Choice of camera	• Because all ports are 10–12 mm, there is no need for a 5-mm laparoscope; most surgeons use a 10-mm laparoscope, which offers better optics. • A 30° laparoscope is preferred. • Use of right-angle light source adapter & bariatric length laparoscope decreases external collisions.
Choice of energy device^a^	• Higher degree of articulation results in improved tissue manipulation (complete 360° rotation without restriction is ideal). • Bariatric length instruments enable surgeon to stagger instruments externally & avoid external collisions. • Tip needs to cool quickly. • Device needs to be a good grasper & dissector. • Avoid ultrasonic devices, as tension is required at the time of vascular pedicle transection during a hysterectomy, which induces poor hemostasis
Suction device	• Laparoscopic suction is not needed for a vNOTES hysterectomy. • Place Yankauer suction tip through one of the ports while suction tubing is clamped, & then intermittently clamp & unclamp suction tubing to evacuate the blood while avoiding a loss of pneumoperitoneum. • If more suction is needed, remove the GelSeal^®^ Cap if using GelPOINT V-Path port, suction the field, & replace the GelSeal Cap.

**Vaginal route**	
Hysterectomy progresses from difficult to easy parts of the procedure	• In the vaginal route, a colpotomy is made first, followed by posterior & anterior entry. The vesicovaginal space is typically developed first, then the uterine arteries are secured. With this approach, the most difficult part of the hysterectomy—mobilizing the bladder, lateralizing the ureters, transecting uterosacral & cardinal ligaments, & securing the uterine arteries—are completed at the beginning of the case. • The surgeon is working from the most anatomically challenging area low (caudad) in the pelvis to the less-challenging area higher (cephalad). • The opposite happens with the transabdominal laparoscopic & robotic approach, wherein the surgeon is working down toward the pelvis from wide to narrow, from high to low, from less-vascular to more-vascular, & from less challenging to more challenging. • “Fast is slow”: because of the vaginal route & use of laparoscopic bipolar device, hysterectomy “bites” are smaller in comparison to traditional vaginal hysterectomy which utilizes large metal clamps such Heaneys. As a result, smaller bites are more hemostatic.
Avoidance of abdominal pathology	• Avoid abdominal wall mesh from prior surgery, adhesions in the mid-& upper abdomen, multiple prior abdominal surgeries, rigid abdomen resulting in limited insufflation (nulliparity, abdominoplasty).

**vNOTES essentials**	
Target anatomy (uterus) is close to the camera & instruments	• In comparison to a vaginal route, with a transabdominal laparoscopic or robotic approach, the camera & instruments need to travel further from the abdomen to the bottom of the pelvis or the sidewall.
Insufflation	• While standard insufflation pressures during transabdominal laparoscopy & robotics typically are ∼15 mm Hg, lower pressures are used during vNOTES (8–10 mm Hg). The rationale for lower pressures is simple: the bony pelvis —where the majority of the work takes place—is a rigid structure & unlike the abdominal wall, does not need to be “insufflated” for adequate visualization.
Bowel manipulation & degree of Trendelenburg position needed to move the bowel out of the pelvis	• A laparotomy pad or a 4 x 4 sponge inserted into the posterior cul-de-sac at the beginning of the case helps bowel retraction & visualization; this is especially important when Trendelenburg positioning is not tolerated by the patient making it not feasible to move the small & large bowel out of the pelvis cephalad with an atraumatic or bowel grasper. • Routine use of the pad or sponge is not required if visualization is adequate; avoiding the pad's use minimizes the risk of adhesion formation.
Uterine manipulation & technique of uterine retraction to maximize exposure to the target tissue	• Uterine manipulator is not needed & is not helpful, as it clutters the space needed for the instruments & camera. • Instead, the surgeon can use an instrument in their medial hand (typically an atraumatic grasper such as a wavy grasper) to push the uterus up (cephalad) & away (from the sidewall, medially), in contrast to conventional vaginal surgery, wherein a surgeon pulls the uterus down (caudad), toward oneself. Retraction of the uterus “up & away,” instead of toward oneself is a unique to vNOTES & serves the function of a uterine manipulater used in transabdominal laparoscopic & robotic approaches. • Both instruments should “walk” in tandem along the target anatomy. For example, the uterus should not be “dropped” or let go of, as that results in loss of visualization. Instead, a grasper & a bipolar device will move along the side of the uterus, taking turns for holding it to push the uterus “up & away.”
Exposure & retraction	• The Alexis^®^ Retractor of the vNOTES port serves the function of vaginal retractors (such as a long-weighted speculum, a narrow Deaver, a narrow long-angle retractor, & Briesky retractors). This functionality explains the benefits of vNOTES, such as eliminating the need for skilled assistants experienced in vaginal retraction & exposure during the laparoscopic portion of the procedure. • Surgeon needs to ensure that viscera (bowel & bladder) are far enough away from the site of transection. A laparotomy sponge in the posterior cul-de-sac retracts & bowel & creates a backdrop for upper pedicles & adnexa. Anterior to the uterus, the surgeon needs to assure bladder & small bowel do not “fall” close to the transection site. If visualization is adequate without the pad or sponge, it is not needed; this helps minimize the risk of adhesions
Need for assistants	• The need for assistants to retract is limited to the vaginal portion of the case, which is significantly shorter, compared to the conventional vaginal hysterectomy. • 1 assistant is required to drive the camera; however, the technique of driving the camera differs in the vNOTES approach, compared to the transabdominal multiport approach. With the latter, the surgeon driving the camera actually directs the actions of the second surgeon holding instruments as they “point” where to go. In vNOTES, the surgeon driving the camera operates in the “assist” & not the “primary surgeon” mode because they follow the instruments of the surgeon holding the instruments, who, in turn, drives the surgery. • As a result, inexperienced surgeons & novices (junior residents, surgical physician assistants, nurses, & scrub technicians) can be trained easily to drive the camera with a short learning curve.
Ergonomic considerations	• Retraction provided by the vNOTES port has the potential to reduce ergonomic strain on both the primary surgeon & the 2 assistants. • Converting most of the hysterectomy procedure to a laparoscopic route from a conventional vaginal approach reduces strain on the primary surgeon & eliminates the strain on 2 more assistants. • The laparoscopic portion is unique & different from transabdominal laparoscopy & robotics, as the surgeon remains in the neutral stance during most of the procedure & is able to move freely. This reduces strain on the neck & shoulders in comparison to robotics & laparoscopy, respectively.
Ability to control blood supply completely before tissue extraction is initiated	• In contrast to conventional vaginal hysterectomy, in cases of enlarged uteri that cannot be removed intact & require in-situ coring & oscillation after securing uterine vessels but before upper pedicles are transected, the vNOTES approach secures & transects all pedicles before the specimen is removed.
Contained tissue extraction	• After vNOTES hysterectomy is completed, a containment bag is placed into the pelvis, the specimen is placed into the bag & the bag is exteriorized, enabling contained tissue extraction to be performed in contrast to the conventional vaginal approach, which causes in-situ uncontained tissue extraction while the upper pedicles are still intact. • In addition, in-bag contained tissue extraction offers the advantage of improved exposure & less of a need for vaginal retractor to protect the sidewall & viscera. • Aside from cases when contained tissue extraction is performed routinely, containment bags can be used during vNOTES surgery when such containment is clinically indicated or desired (example: persistent postmenopausal bleeding).

**vNOTES approach: Advanced techniques**	
C-section scar adhesiolysis	• In cases of C-section scars, or limited vaginal access (postmenopausal or nulliparous cases), the ring can be placed into the posterior cul-de-sac peritoneally but can sit in the anterior fornix (similar to a ring pessary), which then enables continuing dissection vaginally with laparoscopic instruments until the anterior colpotomy can be completed safely. For adnexal surgery, when the uterus is not removed, the vNOTES port is placed into the posterior cul-de-sac, but a smaller ring is utilized. • The laparoscopic portion of vNOTES adhesiolysis enables the surgeon to reach further cephalad for more extensive manipulation, compared to the conventional vaginal route, which enables the surgeon to address more-extensive adhesions & convert transabdominal cases to a vNOTES route. • The inner ring is placed between the colpotomy incision on the vaginal epithelium & C-section scar adhesions & peritoneum. • Utilize a “lateral-to-medial” technique by securing the uterine blood supply first, then lysing adhesions lateral-to-medial to reflect ureters, minimize bleeding, & decrease risk of bladder injury. • The anterior peritoneum appears blue or gray due to its translucency in comparison to the dense white scar. Moreover, the peritoneum displays what is commonly referred to as the “wave” or the “sail” sign because the peritoneum moves in & out slightly from the intermittent insufflation of or patient ventilation. • The surgeon can retrograde fill the bladder, but that is rarely necessary due to the “upside down” view of the bladder, which is retracted by the inner ring.
Cervical elongation	• Similar to the challenge of the C-section scar, which is difficult to reach with a conventional vaginal approach, in cases of cervical elongation, the anterior peritoneal entry can be performed laparoscopically in vNOTES.
Fibroid management	• The uterus can be rotated in the pelvis sequentially into more-favorable orientation to improve exposure as the hysterectomy proceeds. • “easier” side of the hysterectomy can be completed first to detach the uterus from that side to gain access to the more difficult side. • The surgeon must be aware of sidewall proximity during dissection to ensure safe distancing from the ureter & vessels.

^a^ Based on these characteristics, the most commonly used device is the 5 mm LigaSure™ Blunt Tip (Medtronic, Dublin, Ireland). A LigaSure (Maryland, USA & Medtronic) tip can also be used in cases of extensive C-section scar adhesions due to its shape. Applied Medical (Rancho Santa Margarita, CA, USA) manufactures the Voyant^®^ Intelligent Energy System, a popular advanced bipolar energy device in settings where it is available due to its ability to articulate without restriction. Any of the ultrasonic devices (HarmonicACE™ [Ethicon, Raritan, NJ, USA], the Thunderbeat^®^ [Olympus, Center Valley, PA, USA], the Sonocision™ [Medtronic]) are not suitable options, as they need to be activated off the tension, are not designed for grasping & take longer to cool. The Enseal™ (Ethicon) device has a wider jaw that does not seem to work as well in LESS surgery due to space restriction.

LESS, laparoendoscopic single-site surgery; vNOTES: vaginal natural orifice transluminal endoscopic surgery; FOV, field of view; C-section, cesarean section.

**Table 3.** Vaginal Natural Orifice Transluminal Endoscopic Surgery: Hysterectomy Step-by-Step with Tips and Tricks.

Step	Details	Tips & tricks
**Vaginal part**		
1. Perform colpotomy & transection of uterosacral ligaments	• Circumferential incision of vaginal epithelium • Anterior and posterior colpotomy	• Technique is identical to conventional vaginal hysterectomy (circumferential incision around the cervix & anterior & posterior colpotomy) • Perform examination under anesthesia & check uterosacral ligaments & posterior cul-de-sac for signs of endometriosis & adhesions • Clamp & cut & transfix uterosacral ligaments bilaterally; Heaney stich & tagging each ligament is advised to keep pedicles secure
2. Place peritoneal stay sutures by suturing peritoneum to vaginal epithelium anteriorly & posteriorly	• At the time of anterior and posterior colpotomy, pass a suture through peritoneum & vaginal epithelium at 12 and 6 o'clock, respectively, to approximate peritoneum to vaginal epithelium (no need to tie) • Peritoneal stay sutures will keep those 2 layers approximated during inner-ring placement & ensure that the inner ring slides into the peritoneal cavity & does not pop out of the vagina	• Peritoneal stay sutures at 6 and 12 o'clock to facilitate successful intraperitoneal-ring insertion & minimizes chances of preperitoneal ring placement or expulsion • Peritoneal stay sutures could be used to keep the ring in place if it is unstable & by wrapping suture around the ring & securing sutures with a clamp
3. Stretch perineal incision	• Use the back of long forceps with teeth inserted into anterior & posterior peritoneal incisions to stretch the incision to widen it	• Stretching perineal incisions helps facilitate successful peritoneal inner-ring placement, avoids peritoneal “curtain” or obscured view anteriorly, and enables the inner ring to be more open—all of which improves visualization & instrumentation • Running a locking stich to approximate peritoneum to vaginal epithelium is not recommended at the time of entry for hemostasis (except in a case of a very large colpotomy); doing so might narrow the colpotomy, which limits the size of the incision (which, in turn, restricts movements); moreover, it is not necessary for hemostasis because the inner ring will tamponade minor bleeding from the cuff & peritoneum during the laparoscopic part of the case before it can be controlled at the time of cuff closure
**vNOTES port insertion**		
4. Insert Alexis^®^ Retractor	• An Alexis Retractor of GelPOINT V-Path port is shaped differently than transabdominal LESS port equivalents, designed to fit better into the vaginal canal (inner ring is more flexible for fitting into the pelvic cavity & sleeve is designed to take the shape of the vaginal canal)	• Place Alexis Retractor over the instruments that were used to apply traction to the cervix during the colpotomy • Use the introducer to insert the inner ring into the anterior colpotomy site while pulling on the anterior stay suture; introducer is placed over a retractor, such as a narrow Deaver or a right-angle retractor, then retractor is removed & the inner ring is placed into the indentation of the moving part of the introducer & the introducer is pushed into the cavity while the handle is kept in place; it is important to keep the handle in place without moving it out & only move the lower part with the ring in it forward • Keep the inner ring in place to stabilize with an index finger to hold it in place while removing the introducer; note that the ring will remain unstable until it is placed into the posterior & anterior colpotomy sites • Use the introducer to insert the inner ring into the posterior colpotomy site while pulling on the posterior stay suture & remove the introducer • Fold the outer ring inward twice to shorten the sleeve of the Alexis retractor; note that the outer ring does not need to be right against the perineum & be sure not to overtighten it; it “hangs” loose. Folding the sleeve too many times will result in the inner ring “popping” out of the peritoneal cavity; adjustments can be made for long vaginal canals (example: in obesity) when full length of the sleeve is needed & ring does not need to be folded in at all; rarely, the outer ring needs to be unfolded twice from its default arrangement & not folded at all in the packaging to accommodate a long vaginal canal
5. Place a laparotomy pad or a 4 x 4 into posterior cul-de-sac if visualization is not adequate	• Retraction of the large & small bowel out of the pelvis & away from the surgical site • Improve depth perception by creating a “backdrop” behind the nearby structures as a surgeon is transecting • This ensures that that no bowel is “sneaking up” near the tip of the bipolar device, which, in turn, minimizes the need to move the camera	• In cases when is not possible to place the laparotomy pad into the pelvis due to a wide bulky fibroid uterus, a 4 x 4 sponge can be used instead; one has to be careful not to lose it in the abdomen (some surgeons put a suture through it and thread it through one of the ports for easy retrieval at the end of the case) • Depth perception is diminished in LESS surgery, compared to conventional laparoscopy
6. Assemble a GelPOINT V-Path Transvaginal Access Platform^*^	• Sizing: number refers to the diameter of the inner ring (size 9.5 for most hysterectomy cases, size 7 for adnexectomy, & narrow upper vagina & size 11 for large multiparous vagina • The 6-o'clock position is oriented toward the floor, 12 o'clock toward the ceiling & the surgeon's right is 3 o'clock & the surgeon's left is 9 o'clock; smoke evacuation tubing is attached to the port on the GelSeal R Cap at the 1 o'clock position (the mnemonic is “chimney”) & insufflation tubing is placed at the 3 o'clock position • The V-Path LESS port group includes 4 ports, 3 of which are 10 mm & 1 of which is 12 mm • The camera port is typically placed at 6 o'clock & 2 ports for the instruments are placed at 10 and 2 o'clock • A 4th instrument port is typically placed in the middle in case additional instrumentation is needed • After the port is assembled, instruments are removed from the cervix & the GelSeal R Cap is secured to the outer ring via a latching mechanism	• All ports should be placed ∼1 cm away from the edge to minimize the chance of the port colliding with the rigid rim of the GelSeal^®^ Cap • Placing ports too close together might result in more external instrument collisions • The gel of the cap is designed to reseal itself, so ports can be removed &replaced several times if adjustments are needed or if the port is pulled out during an instrument exchange • In cases when uterosacral ligament suspension is performed at the time of hysterectomy, this port is often used for suture management, in which case, a 12-mm port is preferred • Alternatively, surgeons prefer to place a 12-mm port in a 10 o'clock position to facilitate specimen removal (such as tubes, for example) or introduce a 4 x 4 • The gel of the cap is designed to reseal itself; ports can be removed & replaced multiple times if need be • Introducing instruments directly through the gel, bypassing the port altogether (to facilitate suture management during uterosacral ligament suspension for example); however that is considered off-label^a^ use due to the potential risk of dragging small pieces of the gel into the pelvis
**Laparoscopic part**		
7. Transect cardinal ligaments & uterine arteries bilaterally	• Decreases the amount of back bleeding from the uterus • Enables surgeon to avoid the uterus being pulled toward the contralateral side, requiring more manipulation to triangulate target tissue for adequate exposure	• Alternative technique to bilateral cardinal ligament & uterine artery transection is to transect 1 side continuously from the cardinal ligament up to the level of the round ligament; however, this approach might result in the uterus being pulled to the contralateral side; to correct for this asymmetry, more traction might be required to expose ligaments on the contralateral side • Starting on the patient's left side (the most-common configuration for a right-handed surgeon who will have the bipolar device in his/her right hand), the cervix is pushed medially & cephalad to expose the uterine vessels, which are transected with the bipolar device. • “Up and away” technique of cephalad retraction away from the sidewall is utilized
8. Transect remaining attachments	• In case of ovarian conservation, the broad & utero-ovarian ligaments are transected but the round ligament is typically left intact until the right side is detached to stabilize the uterus; next, the surgeon detaches the remaining attachment on the left side & the pelvis is inspected for hemostasis; alternatively, the broad & round ligaments are transected & the utero-ovarian ligament instead of the round ligament is left attached; while this may result in potentially putting tension on a vascular pedicle, transecting the round ligament might be preferred for other reasons	• Salpingectomy can be performed after the utero-ovarian ligament transection & removal *en block* (attached to the uterus), or in a case when mesosalpinx is difficult to visualize, the tube is transected, the uterus is detached fully & then salpingectomy is performed; with the former approach, the tube is stabilized via its proximal attachment & the latter approach, it requires additional triangulation & retraction with the grasper
	• If bilateral salpingo-oophorectomy is performed at the time of hysterectomy, the broad ligament is transected next, followed by visualization of the ureter & infundibulopelvic ligament; next, adnexa is removed *en block* by transecting the round ligament toward the infundibulopelvic ligament; alternatively, the broad & round ligaments are transected & the infundibulopelvic ligament instead of the round ligament is left attached; while this may result in potentially putting tension on a vascular pedicle, transecting the round ligament might be preferred for other reasons	• Salpingo-oophorectomy can be performed after the uterus is detached if *en bloc* excision is not feasible.
9. Disassemble port, remove laparotomy sponge	—	• The pneumoperitoneum is evacuated to decrease the risk of postoperative shoulder pain via standard maneuvers prior to removal of Alexis Retractor. • Abdominal compression might be necessary in cases when abdominal adipose tissue in obese patients traps pneumoperitoneum in the upper abdomen
10. Remove uterus	• In cases when the uterus cannot be removed intact (examples: fibroids & adenomyosis), either contained or uncontained tissue extraction is performed	• While an Alexis Retractor provides exposure, with an uncontained technique the surgeon needs to use additional instrumentation (such as Brieskys & right-angle retractors) to protect the lateral vaginal sidewall, bladder & rectum; surgeon also needs to ensure that viscera (bowels) are not in close proximity to the morcellation site • Long Lahey clamps & long knife handle are useful to use during vaginal-tissue extraction • Conventional vaginal hysterectomy can be used—such as volume-reduction techniques such as coring, wedging &“paper roll” technique; however, because the uterus is free of its attachments, unlike conventional vaginal hysterectomy, with the vNOTES approach, the ExCITE [Extracorporeal C-Incision Tissue Extraction] technique can be utilized
Contained tissue extraction can be utilized if desired or indicated	• vNOTES hysterectomy makes contained tissue extraction possible because the uterus is fully detached • A containment bag is placed into the pelvis, the port is reassembled, the uterus is placed into the bag laparoscopically & the bag is brought toward the GelSeal Cap. The GelSeal Cap is removed, the bag is exteriorized, & the ring is rolled inward; tissue extraction is performed & the containment bag is removed	• A laparoscopic needle driver can be used to grasp the containment bag at 12 o'clock & 6 o'clock, folding it to stabilize it & bringing it toward the introitus before the GelSeal Cap is removed • As tissue extraction continues, the containment bag is rolled inward, which brings the tissue closer to the opening of the bag • A containment bag provides extra retraction to Alexis Retractor of the V-Path port • If more exposure is needed, additional instrumentation (such as Brieskys & right-angle retractors) can be placed inside the containment bag
11. Remove the Alexis Retractor	—	• Roll out the plastic sheath until it is loose & remove the inner ring from the pelvic cavity
12. Complete the rest of the procedure (identical to conventional vaginal hysterectomy)	• Inspect the uterosacral ligaments & uterine artery pedicles for hemostasis • Perform McCall's culdoplasty unless not appropriate clinically • Close the vaginal cuff	—

^a^ Self-assembled vaginal LESS ports made of any small wound retractor, glove & ports can be used off-label in settings where a GelPOINT V-Path Transvaginal Access Platform is not available. Self-assembled vaginal LESS ports and use are beyond the scope of this article.

LESS, laparoendoscopic single-site surgery; vNOTES: vaginal natural orifice transluminal endoscopic surgery.

One challenge of any LESS procedure, including vNOTES, is restricted movements and their sequelae, such as loss of triangulation and limited visualization. While multiport laparoscopic and robotic surgeons are used to manage movement restriction resulting from an instrument confined to a transabdominal port, LESS surgery, including vNOTES, results in unique movement restriction because all instruments are confined to the same port. (Fig. 2A–C).

A nuance specific to vNOTES hysterectomy is that the LESS incision in vNOTES is the vaginal colpotomy. By default, the vaginal colpotomy is going to be a larger, more-flexible, and less rigid incision than the most commonly utilized 2–3 cm transabdominal incision (Fig. 2D–F). Another consideration in vNOTES hysterectomy is that, at the beginning of the case, the inner ring is bent in a narrower configuration, but, as the surgeon transects cardinal ligaments and moves cephalad along the broad ligament, the inner ring opens and unfolds, and restriction is reduced. Yet, as the surgeon moves cephalad along the broad ligament from the distal to the proximal portion of the pelvis, the surgeon moves farther and farther away from the inner ring. Thus, movements become more restrictive again.

Another challenge in LESS surgery in general (including vNOTES) is internal and external instrument collisions and crowding. In vNOTES, to minimize external collisions, one option is to orient the surgeon's hands horizontally rather than vertically which, in turn, requires using articulating instruments (Fig. 3 A, C–E). In addition, using a right-angle light adapter moves the light cord out of the way (Fig. 3B). Yet another is to option is to stagger instruments by using a bariatric length scope and a bariatric length bipolar device (Fig. 3E).

As with transabdominal LESS, the surgeon has 2 options in vNOTES instrument maneuvering: (1) a parallel technique or (2) a cross technique (Fig. 2D–F).^21,22^ When working in parallel, 2 instruments move conventionally as they would in multiport transabdominal laparoscopy; however, when working space is limited, this technique may result also in instrument crowding, and internal and external collisions. Working space in LESS is thought of as an ellipse and its shape is determined by the camera degree, the distance between instruments and the camera, and the length of the instruments. For example, in LESS, if all instruments point to the same target, and do not cross each other with the use of a 0° laparoscope, all instruments are parallel to each other, and the range of movement is limited (Fig. 2B). However, using a 30° lens leads to a laparoscope crossing with the instruments. Furthermore, keeping 1 instrument static for tissue traction provides more space for movement of the second instrument; static and dynamic instruments are alternated as needed (Fig. 2C).

As in LESS, in vNOTES, using a 30° lens expands the view; while few surgeons prefer to use a 0° laparoscope, as it requires less skill to operate, most prefer the advantages of a 30° scope (Fig. 2B, 2D–F). Of note, using a flexible scope has been tried in vNOTES and has not been found useful for addressing this limitation.^5^

Despite the surgical techniques described above, movement restriction remains a challenge for higher-complexity vNOTES cases. For example, procedures that require extensive sidewall dissection (endometriosis, lymph nodes) or suturing (myomectomy) are performed by few surgeons.^7,23–33^ Movement restriction results in a decreased ability to triangulate, which requires surgeons to cross instruments and manage image reversal. Several robotic platforms are being explored to address this limitation.^7,23–29^

Finally, smoke evacuation can affect visualization. In conventional, transabdominal, multiport laparoscopic and robotic surgery, tubing that insufflates the abdomen is typically attached to one of the lateral ports, and tubing that is used for smoke evacuation is attached to another port (usually as far away from the insufflation port as possible and away from the camera port). In any LESS surgery, insufflation and smoke evaluation ports are located in close vicinity (in the gel cap). Those challenges tend to be less problematic in vNOTES in comparison to transabdominal LESS surgery, as smoke tends to partially float cephalad out of the pelvis, away from the surgical field.^5^

In cases when the uterus cannot be removed intact, conventional volume-reduction techniques (coring and wedging) and ExCITE (extracorporeal C-incision tissue extraction) techniques can be utilized or contained in-bag tissue extraction is performed if indicated or desired.^34–40^ In vNOTES, unlike in conventional vaginal hysterectomy, all blood supply is secured as the uterus can be rotated easily in the pelvis in cases of patients with enlarged uteri before specimen removal (Video 1).

In addition to conserving costly human resources, retraction provided by the vNOTES port has the potential to reduce ergonomic strain on both the primary surgeon and the 2 assistants.^41–44^ Studies need to compare surgeon ergonomics between the different routes.

## Video 1.

Vaginal natural orifice transluminal endoscopic surgery hysterectomy for large fibroid uterus and ureteral course.

## Video 2.

Cesarean-section scar adhesiolysis and laparoscopic anterior entry in cervical elongation during vaginal natural orifice transluminal endoscopic surgery hysterectomy.

## Video 3.

Vaginal natural orifice transluminal endoscopic surgery hysterectomy, part 1. (vaginal).

## Video 4.

Vaginal natural orifice transluminal endoscopic surgery hysterectomy, part 2. (laparoscopic).

## Video 5.

Vaginal natural orifice transluminal endoscopic surgery port assembly.

## Video 6.

Vaginal natural orifice transluminal endoscopic surgery hysterectomy set-up tips.

## Video 7.

Vaginal natural orifice transluminal endoscopic surgery hysterectomy surgical techniques.

## Video 8.

Bilateral salpingo-oophorectomy at the time of vaginal natural orifice transluminal endoscopic surgery hysterectomy.

## Postoperative Care

vNOTES hysterectomies are ideal candidates for SDS (same day surgery) in ambulatory surgical centers with aggressive use of enhanced-recovery pathways.^45^ In comparison to the transabdominal laparoscopic approach, avoiding abdominal ports helps decrease postoperative pain. In comparison to the vaginal route, patients are exposed to pneumoperitoneum; however, it is easier to evacuate the gas at the end of the procedure due to the larger aperture of the vNOTES port. Furthermore, avoidance of metal vaginal retractors, and difference in handling of the pedicles (clamping, cutting, and tying versus using advanced bipolar devices) could potentially explain why patients who undergo vNOTES might experience less pain, compared to patients who undergo conventional vaginal hysterectomy. Unfortunately, studies on this topic do not address questions about postoperative pain adequately, as that metric is not the primary endpoint of most studies. As a result, some vNOTES studies show lower pain scores,^46^ while others do not^47^; a systematic review and meta-analysis comparing vNOTES to laparoscopic hysterectomy published in 2020 showed no difference in pain scores after vNOTES hysterectomy.^48^

Finally, this topic is difficult to study as ERAS (enhanced recovery after surgery) protocols involve many components and vary widely from institution to institution. Based on clinical experience alone, many vNOTES surgeons note a decrease in pain scores after vNOTES hysterectomy in comparison to other routes and adjust their ERAS protocols and preoperative counseling accordingly.

## What We Know So Far—the Evidence-Based vNOTES Hysterectomy

The concept of transvaginal endoscopic surgery is not new—it was explored by Semm in the 1960s. Unfortunately, at that time, laparoscopic equipment such as cameras, instruments, and light sources were not adequate to develop the technique. Three decades later, transvaginal hydrolaparoscopy was developed as a diagnostic procedure and is considered a precursor for vNOTES.^49^ In the 2000s, vaginal natural orifice surgery was first reported in general surgery for cholecystectomy and appendectomy.^50,51^ A decade later, surgeon–innovators published their work on vNOTES hysterectomy. MIGS surgeons in Taiwan, under the leadership of Chyi-Long Lee, shared their experience by publishing the first feasibility study describing NOTES hysterectomies, utilizing a self-made port made of a glove and a small wound retractor.^52^

At the same time, Jan Baekelandt in Belgium utilized a similar technique and became not only a vNOTES pioneer in the Western world^53^ but also a surgeon–scientist whose team researched and published extensively during that early innovation and later development phases following the principles of the IDEAL guidelines.^54^ He and his team first documented complication rates in vNOTES hysterectomies in a large series^8^ that spanned 5 years during this early adoption phase (2013–2018) involving 1000 vNOTES surgery patients, of which 73% were hysterectomies. The team noted a 5.2% complication rate (1.4% intraoperative and 3.8% postoperative). Most of the intraoperative complications involved cystotomies (9 cases; 1.2%). The mean specimen weight was 172 g (range: 20–3361 g), and the average operating time was 46 minutes (range: 20–250 minutes).

While descriptive studies are a great starting point for understanding innovation and setting the stage for future research, ultimately, randomized controlled trials (RCTs) are needed to understand how to utilize new approaches in a way that would benefit patients. There are multiple challenges to conducting high-quality research in MIGS, one of which is low complication rates.

Keeping that in mind, Baekeland and colleagues conducted a noninferiority RCT comparing vNOTES to the transabdominal laparoscopic approach for benign hysterectomy patients powered for conversions from vNOTES route, HALON (hysterectomy by transabdominal laparoscopy or natural orifice transluminal endoscopic surgery). The authors randomized 70 patients to vNOTES hysterectomy with sham skin incisions or laparoscopic hysterectomy (ClinicalTrials.gov NCT02631837).^47^ The researchers concluded that vNOTES hysterectomy was noninferior to laparoscopic hysterectomy because neither group had any conversions; however, when looking at secondary outcomes, the vNOTES arm had a shorter length of stay and left the hospital earlier. Postoperative complication rates did not differ between the groups.

A systematic review and meta-analysis comparing vNOTES to laparoscopic hysterectomy published in 2020 included the RCT described above and 5 retrospective studies.^48^ The researchers found that vNOTES had shorter operative times and lengths of stay, and lower estimated blood losses, with no differences in complications, readmissions, and pain scores. As utilization of vNOTES hysterectomy increased, surgical repertoires expanded to higher-complexity cases, a trend reflected in publications.

Several retrospective studies described the use of vNOTES hysterectomies in different settings, all with similar favorable safety profiles: a retrospective review of large uteri with a mean weight of 559 ± 425 g (range: 281–3361g)^55^; uteri >1 kg^10^; obesity^56,57^; virginal patients^9^; and gender-affirming hysterectomy.^58^ A vaginal NOTES hysterectomy has been described wherein the entire procedure was completed laparoscopically and could be explored further in cases when the vaginal portion is the most challenging.^53^ A large international multicenter RCT is currently underway to compare vNOTES, conventional vaginal, and laparoscopic hysterectomies (the VANOLAH trial; ClinicalTrials.gov NCT05971875).

Tracking vNOTES cases in the United States is problematic because no Current Procedural Terminology code exists for vNOTES. Most surgeons use laparoscopically assisted vaginal hysterectomy codes for vNOTES hysterectomy when billing, which complicates tracking. The International NOTES Society has a voluntary case registry, but these data are not available for public use.^59^

Future studies are needed to understand the cost of vNOTES hysterectomies, compared to other routes, patient preferences, surgeon factors, training, implementation, and adoption factors.

## Conclusions

vNOTES is not a new surgical technique or tool, but rather a new surgical approach that combines and modifies several existing techniques. The current authors hope that it will allow us to expand the range of surgical repertoire in MIGS, which, in turn, will benefit patients.
